# Lp(a) in daily clinical routine: risk-factor for both cardiovascular events and heart-failure? A retrospective analysis of the Luebeck Lp(a) heart-failure (HF) registry in patients after myocardial infarction

**DOI:** 10.1016/j.athplu.2025.07.002

**Published:** 2025-07-12

**Authors:** Matthias Mezger, Tilmann Solle, Dominik Jurczyk, Caroline Fatum, Felicitas Lemmer, Ingo Eitel, Christina Paitazoglou

**Affiliations:** a Department of Cardiology, Angiology and Intensive Care Medicine, University Heart Center Lübeck, Lübeck, Germany; b German Center for Cardiovascular Research (DZHK), Partner Site Hamburg/Kiel/Lübeck, Lübeck, Germany

**Keywords:** Lp(a), Heart-failure, Myocardial infarction, Pelacarsen

## Abstract

**Background and aims:**

Atherosclerotic cardiovascular disease (ASCVD) is a major health burden being the leading cause of death in Europe. Lipoprotein (a) (Lp(a)) is an important risk factor for CV events reflected by the 2019 ESC recommendation of a once in a lifetime Lp(a) measurement. Furthermore, heart-failure (HF) is the number one diagnosis for hospital admission in Germany and Europe. HF and ASCVD share common well-known risk factors, e.g. diabetes, obesity and hypertension. So far, there is scarcity of data regarding the relationship between Lp(a) and HF. We hypothesized that Lp(a) might be elevated in a high-risk ASCVD patient collective and that there might also be an association with heart-failure.

**Methods:**

The Luebeck Lp(a) HF registry is a combined retrospective/prospective single-center, all-comers registry which investigates the relationship between Lp(a) and HF. The retrospective analysis reported here, comprises patients who were admitted to our heart-catheterization laboratory in the year 2021 due to ST-segment elevation myocardial infarction (STEMI) or non-ST-segment elevation myocardial infarction (NSTEMI).

**Results:**

We found that Lp(a) was assessed only in a minority of patients presenting with STEMI (33 %) and NSTEMI (14.6 %), *p* < 0.001. There was no relationship between Lp(a) level and ejection fraction (EF) or NTproBNP as surrogate markers for HF, respectively. Statin pretreatment was more frequent in patients with NSTEMI (31.1 %) compared to STEMI patients (11.3 %), *p* < 0.001.

**Conclusion:**

Despite ESC recommendation, routine Lp(a) measurement is only rarely performed even in a high-risk patient collective. In patients with MI, we could retrospectively not observe a correlation between Lp(a) levels and heart failure, as assessed by surrogate markers as EF and NTproBNP.

## Highlights

• Lp(a) was rarely measured in clinical practice in high-risk ASCD patients.
• LV-EF and NTproBNP did not correlate with Lp(a) levels.
• Statin pretreatment was more frequent in STEMI compared to NSTEMI patients.

## Graphical abstract

**Figure:**
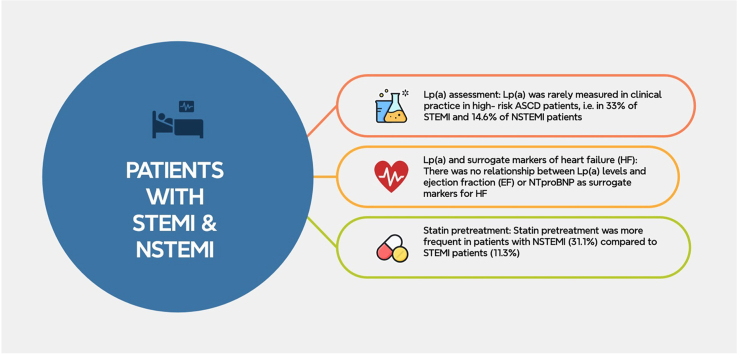

## Abbreviations list:

*ASCVD*: Atherosclerotic cardiovascular disease
*HF*: Heart failure
*NSTEMI*: non-ST-segment elevation myocardial infarction
*STEMI*: ST-segment elevation myocardial infarction
*MI*: Myocardial infarction
*Lp(a)*: Lipoprotein (a)
*CV*: Cardiovascular
*DM-2*: Type 2 diabetes mellitus
*UA*: Unstable angina
*IQR*: interquartile range

## 1 Introduction

Atherosclerotic cardiovascular disease (ASCVD) is responsible for 45 % of deaths in Europe, therefore, still being the leading cause of death despite many improvements in diagnosis and treatment [1]. Besides “classical” cardiovascular (CV) risk-factors, i.e. hypertension, smoking or elevated low-density lipoprotein (LDL-C) cholesterol, also Lipoprotein (a) (Lp(a)) has been associated with CV events, especially coronary revascularization in case of significantly elevated levels (Lp(a) > 150 nmol/l) [2]. Indeed, the 2019 ESC guideline for the management of dyslipidemias give a class IIa/Level of evidence C recommendation for a “once in a lifetime” Lp(a) measurement, with a comparable CV risk-profile of patients with elevated Lp(a) (>180 mg/dl and >430 nmol/l, respectively) to patients with heterozygous familial hypercholesterolemia [3]. Furthermore, the ESC launched a consensus statement in 2022 further discussing the relevance of Lp(a) as an independent and strong CV- risk factor and giving advice for medical professionals [4].

Heart failure (HF) is a global epidemic and the number one diagnosis for hospital admission in Germany and Europe [5]. Globally, 64 million people are living with HF (meaning 1–2 % of the global population) [6]. Due to increasing numbers of people suffering from type 2 diabetes mellitus (DM-2), obesity and hypertension [7,8] and improved treatment of myocardial infarction, the amount of HF patients is expected to rise in the next decades. Furthermore, there was an almost linear increase observed in the individual CV- risk with rising Lp(a) levels [9]. The association of Lp(a) elevation and coronary artery disease [10], peripheral artery disease and calcifying aortic valve stenosis is well established [11]. Today, there is still a scarcity of data regarding elevated Lp(a) and HF, however, several studies and a meta-analysis point towards an association of elevated Lp(a) and the development of HF especially in patients with high Lp(a) values [[11], [12], [13]].

## 2 Patients and methods

### 2.1 Study design

The Luebeck Lp(a) HF registry is a combined retrospective/prospective single-center, all-comers registry performed at our site (University Heart Center Luebeck (UHZL), Luebeck, Germany). The UHZL is an advanced HF- center within a heart-failure network with admission of HF- patients from the whole state of Schleswig-Holstein in the northern part of Germany.

The aim of the Lp(a) HF registry is to systematically investigate the role of Lp(a) for the development of HF since we hypothesized that Lp(a) might be elevated in a high-risk ASCVD patient collective and that there might also be an association with heart-failure.

As a first step, a retrospective analysis of those patients who were admitted to our heart-catheterization laboratory in the year 2021 due to ST-segment elevation myocardial infarction (STEMI) or non-ST-segment elevation myocardial infarction (NSTEMI) was done to uncover the prevalence and relevance of elevated Lp(a) in an ASCVD high-risk patient collective. Lp(a) was analyzed from freshly taken blood samples during index hospital admission. Patients with unstable angina (UA) were excluded (although UA, besides NSTEMI and STEMI, also belongs to acute coronary syndromes) since we expected the highest HF-risk in the patients with STEMI and NSTEMI. Data were collected and analyzed from inhospital patient electronic databases after obtaining approval from ethics committee (22–163). Due to the retrospective character of the study no informed consent from individual patients needed to be obtained. In parallel, we began to do routine measurement of Lp(a) in all patients presenting to our heart-failure outpatient clinic who were asked to participate in our Lp(a)- HF- prospective analysis. Currently, the prospective analysis is still ongoing.

Here, we report the retrospective analysis, which comprises the patients who were admitted to our tertiary-care hospital heart-catheterization laboratory in the year 2021 with either the diagnosis STEMI, n = 158 patients or NSTEMI, n = 369 patients. The main aim of the retrospective analysis was to describe the prevalence of Lp(a) assessment in clinical routine in a patient collective with myocardial infarction as index event and whether there is a relationship between Lp(a) levels and heart failure, as assessed by surrogate markers as EF and NTproBNP. Particularly patients with very high Lp(a) levels (>150 nmol/l) were analyzed.

### 2.2 Statistical analysis

Statistical analysis was performed with IBM SPSS (V. 29)**.** A two-sided *p-*value of *< 0.05* was chosen for statistical significance. Categorial variables were analyzed with Chi- Square and Fishers’ exact test when statistically appropriate. Mann- Whitney- U- test was used for comparison of continuous variables which were displayed as median and interquartile range (IQR, Q1, Q3). Normal distribution was tested with Kolmogorow-Smirnow-test.

## 3 Results

We identified 527 consecutive patients, treated by catheterization at the UHZL in 2021 with the diagnosis STEMI (n = 158) and NSTEMI (n = 369) (Table 1). STEMI patients were younger than NSTEMI patients (STEMI vs. NSTEMI: 70 years (59.0, 78.7) vs. 73.3 years (64.0, 81.8), *p* = 0.004), more frequently smoker (STEMI vs. NSTEMI: 36.7 % vs. 25.2 %, *p* = 0.025) and STEMI patients had more often a family history of myocardial infarction (STEMI vs. NSTEMI: 17 % vs. 8 %, *p* = 0.02). Left-ventricular function as assessed by ejection-fraction (EF) measurement with echocardiography at day 3–4 after catheterization (STEMI vs. NSTEMI: 48 % (37, 55) vs. 50 % (38, 55), *p* = 0.375) and HF biomarker levels (NT-proBNP 1486 pg/ml (386, 4854) vs. 2237 pg/ml (594, 8750), *p* = 0.182) were comparable between both groups.

**Table 1:** Baseline characteristics.

	STEMI (N = 158)	NSTEMI (N = 369)	∗p
male	112 (70.8 %)	229 (62.0 %)	
age	**70.0 (59.0–78.7)**	**73.5 (64.0–81.3)**	**0.004**
height (cm)	176 (168–180)	173 (165–180)	
weight (Kg)	83.0 (74.0–92.0)	80.0 (70.0–92.0)	
BMI	26.8 (24.7–29.7)	26.8 (24.3–30.4)	
smoking	**58 (36.7 %)**	**93 (25.2 %)**	**0.025**
hypertension	88 (55.6 %)	224 (60.7 %)	
type 2 Diabetes Mellitus	32 (20.2 %)	94 (25.4 %)	
hypercholesterolemia	55 (34.8 %)	106 (28.7 %)	
family history for CV disease	**27 (17.0 %)**	**33 (8 %)**	**0.02**
Coronary artery disease	47 (29.7 %)	136 (36.8 %)	
peripheral vascular disease	8 (5.0 %)	36 (9.7 %)	
cerebrovascular disease	13 (8.2 %)	27 (7.3 %)	
atrial fibrillation	19 (12.0 %)	69 (18.6 %)	
preexisting heart failure	10 (6.3 %)	47 (12.7 %)	
EF (%) index- event	48 % (37–55)	50 % (38–55)	
creatinine (μmol/l)	88 (74–118)	92 (74–123)	
GFR (ml/min/m2)	75 (52–93)	66 (45–85)	
LDL (mmol/l)	3.0 (2,2–3,9)	2.7 (1,9-3,5)	
HDL (mmol/l)	1.1 (0,8-1,3)	1.1 (0,9-1,4)	
total cholesterol (mmol/l)	4.6 (3.8–5.4)	4.3 (3.3–5:3)	
Statin pretreatment	**18 (11.3 %)**	**115 (31.1 %)**	**<0.001**
NTproBNP (pg/ml)	1486 (386–4854)	2237 (594–8750)	
Troponin max (ng/l)	**1394 (151–4473)**	**156 (62,3–529)**	**<0.001**
CK max. (U/l)	**1052 (376–2829)**	**173 (90–475)**	**<0.001**
CK-MB max. (U/l)	**107 (57–270)**	**45.8 (24.2–110)**	**<0.001**
Hb (g/dl)	**13.7 (12.2–14.9)**	**12.9 (11.1–14.4)**	**0,007**
Lp(a) measured on index- event	**52 (33 %)**	**49 (13.2 %)**	**<0.001**
Lp(a) index- event (nmol/l)	41 (16–139)	36 (17–104)	
Lp(a) measured within a year after the index- event	1 (0.6 %)	11 (3.3 %)	
Lp(a) known from medical history	5 (3.2 %)	10 (3.0 %)	
cardiogenic shock	**52 (33 %)**	**39 (10.6 %)**	**<0.001**
death during PCI	**6 (3.8 %)**	**2 (0.5 %)**	**<0.001**
death during hospital stay	**34 (21.5 %)**	**24 (6.5 %)**	**<0.001**
duration of hospital stay	5 (4–9)	5 (3–8)	

However, Lp(a) was assessed more frequently in STEMI than NSTEMI patients (33 % vs. 14.6 %, *p* < 0.001), albeit a low frequency of Lp(a) assessment in both cohorts of <50 % in-hospital during the index event could be seen. In 3.2 % STEMI- and in 3 % NSTEMI patients, Lp(a) had been analyzed before the index event. Lp(a) levels were low and comparable between both groups (STEMI vs. NSTEMI: 41 nmol/l (16, 139) vs. 36 nmol/l (17, 104), *p* = 0.884). Regarding parameters describing the extent of myocardial infarction we saw significantly higher values in patients with STEMI compared to NSTEMI patients: Troponin (STEMI vs. NSTEMI: 1394 ng/l (151, 4473) vs. 156 ng/l (62.3, 529), *p* < 0.001), creatinine kinase (CK) (STEMI vs. NSTEMI: CK 1052 U/l (376, 2829) vs. 173 U/l (90, 475), *p* < 0.001, CK-MB (STEMI vs. NSTEMI: 107 U/l (57, 270) vs. 45.8 U/l (24.2, 110), *p* < 0.001). Additionally, a significantly higher proportion of patients with STEMI suffered from cardiogenic shock (STEMI vs. NSTEMI: 32.9 % vs. 10.6 %, *p* < 0.001), death during PCI (STEMI vs. NSTEMI: 3.8 % vs. 0.5 %, *p* < 0.001) and death during index hospital admission (STEMI vs. NSTEMI: 21.5 % vs. 6.5 %, *p* < 0.001). However, no difference between both groups with respect to the duration of the hospital stay was observed (STEMI vs. NSTEMI 5 days (4,9) vs. 5 days (3,8), *p* = 0.818).

**Fig. 1:**
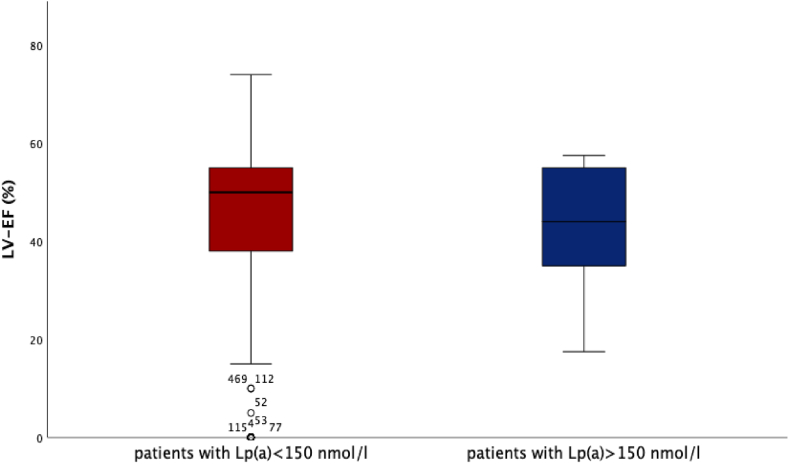
Lp(a) and LV-EF LV-EF was recorded and compared between patients (STEMI & NSTEMI) with Lp(a) < 150 nmol/l (n = 509 patients) and >150 nmol/l (n = 18 patients), showing no difference between both patient collectives: LV-EF for patients with Lp(a) > 150 nmol/l was 44 % (35, 55) and for patients with Lp(a) < 150 nmol/l 50 % (38, 55), respectively, p = 0.252.

**Fig. 2:**
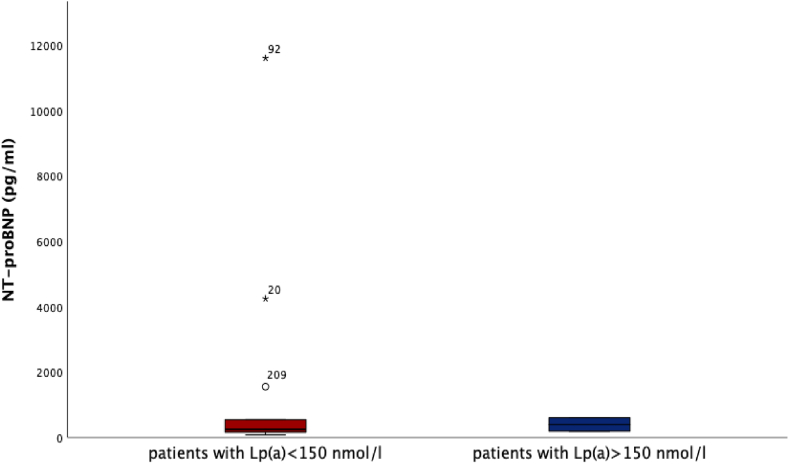
Lp(a) and NT-proBNP NT-proBNP was analyzed for patients with Lp(a) > 150 nmol/l (n = 18 patients) vs. Lp(a) < 150 nmol/l (n = 509 patients). No difference regarding NT-proBNP levels could be detected: NT-proBNP for patients with Lp(a) > 150 nmol/l was 2018 pg/ml (379, 3545) compared to 2054 pg/ml (535, 7879) for patients with Lp(a) < 150 nmol/l, p = 0.331.

We could find very high Lp(a) levels (>150 nmol/l) in n = 18 patients with levels between 152 nmol/l and 386 nmol/l. Lp(a) levels <150 nmol/l were recorded in n = 509 patients. We could neither see a correlation between Lp(a) elevation and left-ventricular function, nor could we observe differences regarding left-ventricular function in patients with very high Lp(a) values (>150 nmol/l) compared to patients with lower Lp(a) values (Fig. 1) (Lp(a) > 150 nmol/l vs. Lp(a) < 150 nmol/l: 44 % (35, 55) vs. 50 % (38, 55), *p* = 0.252). Also, no difference regarding the level of NT-proBNP and Lp(a) levels could be detected (Fig. 2) (Lp(a) > 150 nmol/l vs. Lp(a) < 150 nmol/l: NT-proBNP 2018 pg/ml (379, 3545) vs. 2054 pg/ml (535, 7879), *p* = 0.331).

Of notice, significantly more patients with NSTEMI compared to STEMI patients had statin treatment initiated before the index event (STEMI vs. NSTEMI: 11.3 % vs. 31.1 %, *p* < 0.001). Patients receiving statin therapy exhibited significantly lower LDL cholesterol levels (no statin vs. low- and high-intensity statin: 3.0 mmol/l vs 1.9/2.1 mmol/l, ∗p < 0.001). Additionally, there was a positive, albeit weak, correlation of LDL levels with both high- and low-intensity statin therapy (r = 0.28, p < 0.001). Conversely, no difference was observed between patient groups on high- or low-intensity statins concerning surrogate markers of heart failure (EF and NTproBNP, respectively). Moreover, while NTproBNP did not show any significant correlation, a weak but significant correlation was noted between EF and high-intensity statins (r = 0.13, p = 0.014).

## 4 Discussion

Lp(a) is a known CV risk factor, it has been associated with CV events, but to date no clear relationship to the syndrome of HF has been described. The Luebeck Lp(a) HF registry, a combined retrospective/prospective registry aimed to assess the role of Lp(a) in the development of HF, retrospectively related to patients with a CV event and prospectively independently of clinically manifest ASCVD in HF patients across the whole spectrum of ejection fraction.

Herein, we describe the retrospective collective. We found, that Lp(a) was assessed only in a minority of patients presenting with STEMI and NSTEMI. Furthermore, we could see no relationship between Lp(a) level and EF or NTproBNP as surrogate markers for HF, respectively. In addition, statin pretreatment could be observed more frequently in patients with NSTEMI compared to STEMI patients, possibly due to more NSTEMI patients with already preexisting coronary artery disease and peripheral artery disease. Moreover, a weak but significant correlation was noted between EF and high-intensity statins.

### 4.1 Standardizing Lp(a) assessment is essential

An elevation of Lp(a) particularly in younger patients suffering from MI (incident MI < 50 years) was demonstrated with radioimmunoassay back in the 70ties [14]. Lp(a) is an LDL like particle consisting of apolipoprotein B100 (apoB) linked to apolipoprotein (a) (apo(a)) surrounding a cholesterylester core that builds the LDL component of the particle [15]. Serum concentration and therefore, CV- risk depends on allelic LPA variants responsible for Kringle IV type (KIV) 2 repeat sequences with more repeats meaning slower synthesis and secretion of Lp(a) [16]. Additionally, single-nucleotide polymorphisms (SNPs) and other factors also have an influence on Lp(a) levels [4,17].

Today, most biochemistry laboratories can perform routine Lp(a) measurements. Nevertheless, there is still room for improvement regarding standardization of Lp(a) measurement and reporting. The ESC consensus statement [15] recommends reporting in nmol/l and judgement of cardiovascular clinical risk in laboratory reports for clinicians in minor, moderate, high, very high risk [18]. To enhance and improve research on Lp(a), the European Society of Cardiology published a review in January 2024 highlighting challenges that observational Lp(a) research poses and suggesting requirements to enhance the quality and harmonize Lp(a) data [19].

### 4.2 Lp(a) in clinical routine

Despite clinical availability broader measurements of Lp(a) are not performed routinely. A retrospective healthcare claims study comprising 4 million patient records between 2015 and 2018 in Germany revealed that only 0.25 % of the patients in 2015 and 0.34 % in 2018 received Lp(a) testing [20]. In addition, Lp(a) testing was more frequent either in younger women or in men suffering from atherosclerotic cardiovascular disease (ASCVD) compared to the general population [20]. Our results showed a very limited proportion of patients with Lp(a) analysis before an index event of a myocardial infarction despite guideline recommendations: 3.2 % of the patients with STEMI and 3 % of the patients with NSTEMI. In the general population, according to the Copenhagen General Population Study, 80 % have Lp(a) levels below 50 mg/dl [21]. In addition, a multicenter longitudinal study of midlife aging women in the US, comprising people of different ethnical background, reported lowest Lp(a) values for white people (median and interquartile range: 12 mg/dl and 5–32 mg/dl, respectively) and highest values for African Americans (median and interquartile range: 39 mg/dl and 16–69 mg/dl, respectively) [22]. Regarding specialized cardiovascular care, a retrospective study performed at a cardiovascular center in Germany showed that particularly very high Lp(a) concentrations (>98 mg/dl) were more prevalent at a cardiovascular center (4.6 %) compared to the general population (<0.3 %) [23]. Recently, a non-interventional registry study conducted in German cardiac rehabilitation clinics containing 1351 patients with myocardial infarction <60 years of age demonstrated, that Lp(a) was analyzed only in 4.96 % of these patients. Furthermore, Lp(a) levels above 50 mg/dl and 125 nmol/l, respectively were found in 29.9 % of the patients underlining the importance of Lp(a) testing [24]. In our registry, we retrospectively observed Lp(a)assessment in 33 % of STEMI and 13.2 % of NSTEMI patients during index hospital admission at our tertiary care center in 2021. These findings underline, that we are far away from the 2019 ESC recommendation of a “once in a lifetime” measurement of Lp(a) for everyone, even in a subgroup of patients with a high CV- risk. The more frequent assessment of Lp(a) in STEMI patients observed compared to NSTEMI might be due to the younger age of the STEMI patients and more patients with a family history of MI. We expect, with the increasing focus of the German government and European guidelines on CV prevention a higher proportion of patients undergoing an assessment of Lpa before a CV event or manifestation of ASCVD in the near future.

### 4.3 Lp(a) and heart-failure

There are many class I/level of Evidence A recommendations for HF treatment to improve outcome [25,26]. However, prognosis is still limited and comparable to malignant diseases [27]. The role of Lp(a) in the development or patient risk assessment of HF is not well defined to date. Recently, an association of elevated Lp(a) and incident HF has been discussed especially in patients with high Lp(a) values [12,13]. This effect might be independent from the effect Lp(a) has on atherosclerosis and aortic valve stenosis [11]. An analysis of the Atherosclerosis Risk in Communities Study (including 14.154 study participants) focusing on Lp(a), and HF demonstrated an increased HF risk in patients with highest Lp(a) values (Lp(a) between 23.1 mg/dl and 108.23 mg/dl, respectively) compared to the patients with lowest Lp(a) values (Lp(a) between 0.02 mg/dl and 2.41 mg/dl, respectively) [28]. The risk persisted after adjusting for other CV- risk factors but significance was lost when patients with prevalent and incident myocardial infarction were excluded [28]. This risk was shown to be at least partially mediated by myocardial infarction and aortic valve stenosis according to data from the Copenhagen City Heart and Copenhagen General Population study, comprising 98.097 patients [29]. Also, an increased risk for recurrent HF has been described for patients with elevated Lp(a) levels although the study was small (309 patients), and no echocardiography data were provided [30].

In our retrospective analysis of patients with myocardial infarction, we could neither see a correlation between Lp(a) elevation and left-ventricular function, nor could we observe differences regarding left-ventricular function in patients with very high Lp(a) values (>150 nmol/l) compared to patients with Lp(a) values in a normal range. To further assess the relationship between Lpa and HF, a prospective analysis of stable HF patients presenting in the outpatient clinic across the whole spectrum of EF is ongoing. Patient recruitment has been finished and one-year- clinical follow-up in our outpatient department will be accomplished until 12/2024.

### 4.4 Future perspectives

Currently, there are no specific drugs available that specifically target Lp(a) and lower Lp(a) levels [31]. Statins have been shown to significantly decrease LDL levels and improve outcome [32]. However, regarding Lp(a) levels, even an increase of LDL has been shown [31]. In contrast, PCSK-9 inhibitors, a subcutaneously applied class of drugs was able to show both, a relevant decrease of circulating LDL levels and a decrease of Lp(a) [31]. Results from two running clinical trials testing novel pharmacotherapies that specifically can lower Lp(a) are expected in the next two years: The “Assessing the Impact of Lipoprotein(a) Lowering With Pelacarsen (TQJ230) on Major Cardiovascular Events in Patients with CVD (Lp(a)HORIZON)”, *NTC: NCT04023552* trial and the “Olpasiran Trials of Cardiovascular Events and Lipoprotein(a) Reduction”; Ocean(a) trial, *NTC: 05581303*, investigating Olpasiran vs. placebo in patients with elevated Lp(a) (>/ = 200 nmol/l) and a history of atherosclerotic cardiovascular disease. Pelacarsen is an antisense oligonucleotide (ASO) that has shown to significantly reduce Lp(a) levels by 66–92 % in multidose groups [33]. Olpasiran uses a SI (short-interfering)-RNA approach to lower Lp(a) and demonstrated an Lp(a) lowering effect of 71–97 % in a phase 1 trial [34]. In both trials severe heart-failure is only mentioned as exclusion criterion, but not as an endpoint. Therefore, the influence of both substances on heart-failure needs further investigation in the future.

### 4.5 Limitations and strengths

The main limitations of our study are the single-center- retrospective character, and a small number of patients included. Furthermore, the time frame of the patients analyzed was only one year. However, the main strength is that real world data from a cardiovascular tertiary care center are provided and patients with myocardial infarction were analyzed.

## 5 Conclusion

Despite Lp(a) being a well-known and important CV risk-factor, measurement in clinical routine, as recommended in the European Guidelines, was only performed in a minority of patients after a CV event in our cohort. In patients with MI, we could retrospectively not observe a correlation between Lp(a) levels and heart failure, as assessed by surrogate markers as EF and NTproBNP. The role of Lp(a) in stable HF patients without a CV event, needs to be defined in future studies and will be reported in a prospective analysis of the Luebeck Lp(a) registry.

## Author contributions

MM analyzed data and wrote the manuscript, TS collected data, DJ, CF, FL were involved in conceptualization and formal analysis. IE and CP did critical review of the manuscript.

## Financial support

The work was financially supported by grants from Novartis.

## Declaration of competing interest

The authors declare that they have no known competing financial interests or personal relationships that could have appeared to influence the work reported in this paper.
